# Emulation Evaluation of Interior Beam–Column Connections in PC and RC Moment-Resisting Frames

**DOI:** 10.3390/ma16216906

**Published:** 2023-10-27

**Authors:** Min-Su Jo, Hyeong-Gook Kim, Dong-Hwan Kim, Yong-Jun Lee, Sang-Pil Han, Kil-Hee Kim

**Affiliations:** 1 Department of Architectural Engineering, Kongju National University, 1223-24 Seobuk, Cheonan-si 31080, Republic of Korea; msjo1982@kongju.ac.kr (M.-S.J.); anthk1333@kongju.ac.kr (H.-G.K.); kimdh@kongju.ac.kr (D.-H.K.); 2 Future Research Lab, CAMUS E&C, 8 Uisadang-daero, Seoul 07236, Republic of Korea; yjlee@camusenc.com; 3 Department of Fire Safety and Protection Engineering, Sangji University, 83 Sangjidaegil, Wonju-si 26339, Republic of Korea; hsfeel@sangji.ac.kr

**Keywords:** precast concrete, beam–column connection, moment-resisting frame, seismic performance, emulation evaluation

## Abstract

Precast concrete (PC) structures have many advantages, but their use in the construction of middle- to high-rise buildings is limited. The construction of PC structures requires skills in various operations such as transportation, assembly, lifting, and structural soundness. In particular, regarding the seismic design of PC structures, it is necessary to clearly evaluate whether they have the same structural performance and usability as integral RC (cast-in-place) structures. In this paper, an experimental study was conducted to investigate whether PC members can achieve a seismic performance equivalent to that of RC members in beam–column joints, which are representative moment-resisting frames. The main variables are the two types of structural systems (intermediate and special moment-resisting frames) and the design flexural strength ratio of the columns and beams. The experimental and analytical results showed that the seismic performance of the PC specimens was equivalent to that of the RC specimens in terms of strength, stiffness, energy dissipation, and strain distribution, except for the specimen with splice sleeve bond failure of the column reinforcement (poor filling of the internal mortar). In addition, the I series satisfied the present emulation evaluation criteria for special moment-resisting frames of PC structures, confirming the possibility of applying intermediate moment-resisting frames.

## 1. Introduction

Today’s society has experienced remarkable economic growth since the beginning of the Industrial Revolution, but it is also facing a catastrophic situation due to the effects of climate change. Climate change, the primary result of global warming, is caused by the greenhouse effect, on which carbon dioxide (hereinafter “CO_2_”) is known to have the greatest impact [1]. Industries are thus attempting to realize carbon neutrality by using CO_2_ emission reduction policies centered on developed countries. In the construction sector, various efforts are being made to develop cement substitutes, new construction methods, and new resources.

The precast concrete (hereinafter “PC”) construction method involves producing structural members in a factory and assembling them on site; this process shortens the construction period and facilitates site management. In addition, it is being actively applied in line with the trend of improving the productivity of the construction industry using the modularization of buildings [2]. Since PC members are produced in a factory, it is possible to secure accurate dimensions and secure durability by using high-compression-strength concrete. In addition, the implementation of steam curing for early strength development can improve the replacement ratio of ground granulated blast-furnace slag, a byproduct of the steel industry that replaces cement; this overall process will have large environmental and economic effects because it will reduce CO_2_ emissions [3].

Various studies have been conducted on the application of PC construction. Englekirk [4,5] presented the basic concepts for the development of PC ductile moment-resisting frames for implementation in areas prone to large earthquakes. Palmieri et al. [6] proposed a detailed connection method for PC frame members assembled in high-seismic-risk areas. Restrepo et al. [7], based on their experiments and analyses, presented a joint design method for seismic PC frame members. Palmieri et al. [6], Restrepo et al. [7], Ha et al. [8], Guan et al. [9], Alcocer et al. [10], Ochs and Ehsani [11], and Soubra et al. [12] proposed connection performance improvement and design methods for PC structures. In addition, recent studies investigated the seismic performance of the beam–column connections in cast-in-place and precast buildings [13,14,15]. Based on the results of many studies on PC structures, FEMA’s NEHRP was the first to reflect regulations for PC seismic design [16]. In NEHRP, the connections in PC structures are divided into two cases: wet joints and dry joints. Wet connections are considered equivalent to reinforced concrete (RC) structures cast in place; dry connections are recognized as having poorer performance.

The main objective of the research so far has been to improve the performance of PC structures to match the seismic performance of RC structures. For the PC structure to function as a monolithic structural system, the connection must exhibit sufficient strength, with the connection and its surroundings experiencing less than the allowable deformation [17,18]. However, it is very difficult to perfectly match the performance of a PC structure to that of an RC structure given the level of available technology and the economics involved. Therefore, PC structures with a performance above a certain threshold are evaluated as equivalent to RC structures. This concept is called emulation evaluation, which means “the condition in which the PC structure has the same structural performance and use performance as the cast-in-place concrete structure (hereinafter, “RC structure”)”. If a PC structure has the same performance as an RC structure, the PC structure can be designed by applying the same structural system and seismic coefficients applied to the RC structure [19].

The emulation evaluation criteria for representative PC structures are the American Concrete Institute (ACI)’s 374.1-05 [20] code and the Architectural Institute of Japan (AIJ)’s (2002) [21] guideline. The ACI 374.1-05 [20] code states the “acceptance criteria for moment frames based on structural testing and commentary”, providing tests and evaluation items for PC frames in areas with high seismic risk, i.e., PC special moment-resisting frames can be designed as RC special moment-resisting frames. PC structures are considered to have performances equivalent to those of CIP concrete structures when they exhibit the following behavior:

① Before the drift ratio of the specimen reaches the allowable drift ratio, the lateral strength (load or moment) of the specimen must be equal to or greater than the nominal lateral strength (load or moment) *E_n_*.
② The maximum lateral strength, *E_max_*, should not exceed the lateral strength (*λE_n_*) multiplied by the excess factor (*λ*).
③ The third complete hysteresis curve at the critical drift ratio must satisfy the following conditions:
  - It should be 0.75 times the maximum lateral load (*E_max_*) or greater.
  - The relative energy dissipation ratio must be 1/8 or greater.
  - The secant stiffness at 1/10 of the critical drift ratio must be 0.05 times that of the initial stiffness or greater.

Japan’s “AIJ Guidelines for the Design of Structural Precast Concrete Emulating Cast-in-Place Reinforced Concrete [21]” evaluates emulation differently from the ACI 374.1-05 [20] guidelines. According to the AIJ guidelines, the performances of PC structures must be verified by comparing them with those of RC structures. PC structures are considered to have performances equivalent to those of RC structures when they exhibit the following behavior:

① The flexural yielding strength of PC members must be equal to or greater than that of RC members.
② The strength of PC members must be equal to or greater than that of RC members.
③ The strength as measured in the second complete hysteresis curve at the critical drift ratio must remain within 80%.
④ The deformation at flexural yielding of PC members should not differ by more than 20% from that of RC members.

These guidelines evaluate emulation for strong earthquake regions, and there are many existing studies [22,23] on the equivalent seismic performance of PC and RC members in special moment-resisting frames; however, there is a lack of verification studies for the application of equivalence to intermediate moment-resisting frames in moderate and weak earthquake regions.

In this study, cyclic loading tests of the interior (“+”) beam–column joints were performed on moment-resisting frames for strong earthquake regions, as well as for moderate and weak earthquake regions; an experimental study was conducted to determine whether PC members can achieve a seismic performance equivalent to that of RC members. In addition, the performance of PC members was evaluated according to the requirements of the emulation evaluation guidelines.

## 2. Test Program

### 2.1. Specimen and Design Parameters

**Figure 1:**
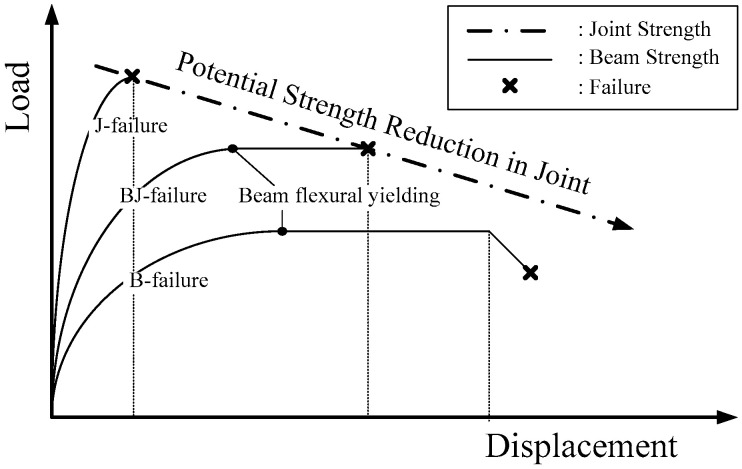
Failure mode of beam–column joint.

In this study, six interior beam–column joints were fabricated. The main variables of the test specimens were the different construction methods (RC or PC), the two types of structural systems (intermediate and special moment-resisting frames), and the design flexural strength ratio of column to beam (*M_c_*/*M_b_*). As shown in Figure 1, beam–column joints can generally be grouped into three failure modes [24]. The first is J-failure, in which the joint fails before the beam adjacent to the column face reaches flexural yield; BJ-failure involves the joint failing after the adjacent beam’s principal reinforcement has yielded and formed a plastic hinge; and finally, in B-failure, the joint remains elastic until bending failure after the beam has developed a plastic hinge. Since the ACI 318-19 [25] and ACI 352R-02 [26] guidelines allow only for BJ or B failure, the six specimens in this study were designed to fail at the plastic hinge of the joint or beam after flexural yielding.

The design of special moment-resisting framing joints according to ACI 318-19 [25] requires a factor of 1.2 (6/5) to be applied to the sum of the beam resistances, such that $\sum M_{c}\geq 6/5\times \sum M_{b}$, where $\sum M_{c}$ and $\sum M_{b}$ are the sum of the design bending strengths of the columns connected to the joint and the sum of the design bending strengths of the beams, respectively. At this time, the stress of the bending tensile reinforcement of the beam at the connection surface should be calculated as 1.25 times the yield strength (*f_y_*). Therefore, 1.2 × 1.25 = 1.5, so the special moment-resisting frame test specimen (hereinafter “S series”) is designed to be 1.63. The ratio $\sum M_{c}/\sum M_{b}$ of the S series is designed to be 1.63. According to ACI 318-19 [25], the ratio *V_jn_*/*V_jby_* of the intermediate moment-resisting frames should be designed to have a value greater than 1.0; because plastic hinges must form at the beams, the ratio *V_jn_*/*V_jby_* of the intermediate moment-resisting frames (hereinafter “I series”) is set to be 1.25. In addition, a variable based on the bending strength ratio of the columns and beams (*M_c_/M_b_*) was applied. For RC-BJ-I and PC-BJ-I, *M_c_/M_b_* was set to 1.65; for PC-BJ2-I, *M_c_/M_b_* was set to 1.24; and for PC-BJ3-I, *M_c_*/*M_b_* was set to 0.98. The entire set of variables of the experiments is summarized in Table 1.

The shear strength of the beam–column joint (*V_jn_*) was calculated using Equation (1) from ACI 318-19 [25].

(1) $$V_{jn}=\lambda_{j}f_{c}^{\prime }A_{j}$$

where *V_jn_* is the joint shear strength, *λ_j_* is the joint confinement factor, *f*′*_c_* is the concrete compressive strength, and *A_j_* is the effective area of the joint. Since the value *λ_j_* used in ACI 318-19 [18] is equivalent to Type 2 in ACI 352R-02 [26], *V_jn_* uses *λ_j_*, which is equivalent to Type 1 in ACI 352R-02 [26].

The joint horizontal shear force, *V_jby_*, at the time of the main reinforcement in the beam yielding was calculated using Equation (2), derived from the equilibrium of the forces acting on the beam–column joint [27].

(2) $$V_{jby}=\left(\frac{l_{c}}{z_{b}}-1\right)V_{by}\frac{l_{b}}{l_{c}}-\frac{h_{c}}{z_{b}}V_{by}$$

where *l_c_* is the height of the column, *z_b_* is the distance between the centers of the top and bottom reinforcing bars of the beam, *l_b_* is the length of the beam, and *h_c_* is the height of the column cross-section. The shear force *V_by_* when the beam yields is calculated using the yield strength of the beam’s tensile reinforcement.

**Figure 2:**
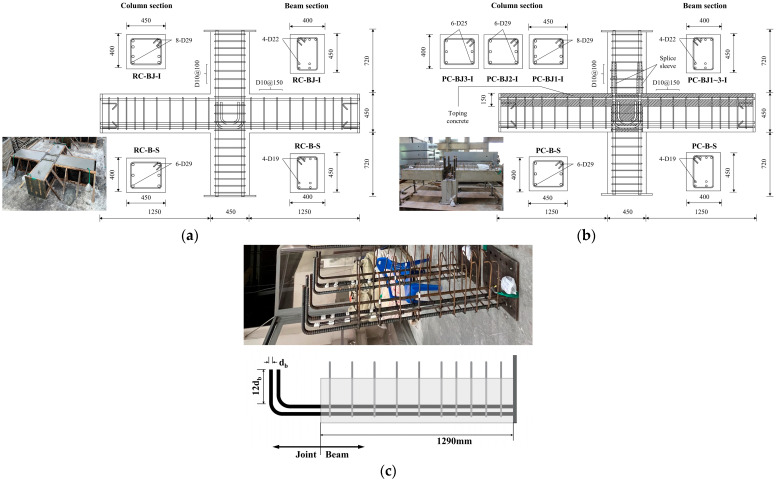
Configurations and reinforcing bar details of test specimens (Unit: mm). (**a**) RC specimen; (**b**) PC specimen; (**c**) Longitudinal reinforcing bars of the PC beams.

The reinforcement details of the beams and columns are shown in Table 2 and Figure 2. The total lengths of the columns and beams in the test case are 1890 mm and 2950 mm, respectively, and the section size is (B_c_)450 mm × (H_c_)400 mm and (B_b_)400 mm × (H_b_)450 mm. For the PC specimen, the beams included 150 mm of topping concrete.

The PC specimen was fabricated using the most common wet PC joining method. The upper, lower, and left and right beams were fabricated separately and connected using 150 mm thick topping concrete and splice sleeves (Figure 2b). The longitudinal rebar of the PC beam was bent into an L-shape and anchored to the column. The lower and upper columns were connected using a splice sleeve with a length of 500 mm, an outer diameter of 76.3 mm, and an inner diameter of 54 mm. After the members were poured, non-shrinkage mortar was injected to consolidate them. The placement of transverse reinforcement inside the joints was in accordance with ACI 318-19 [25] for intermediate moment framing and special moment framing.

### 2.2. Material Specifications

The physical properties of the rebar used in the test are shown in Table 3. The reinforcement for the columns was D29. D22 and D19 were used for the beams. The stirrups and hoops used D10. Concrete was poured once for the columns and beams and twice for the connections. The compressive strength values of the first and second sets of poured concrete were 27.7 MPa and 37.0 MPa, respectively, and the strength of the mortar injected into the splice sleeve was 21.0 MPa.

### 2.3. Test Setup, Instrumentation, and Loading Protocol

**Figure 3:**
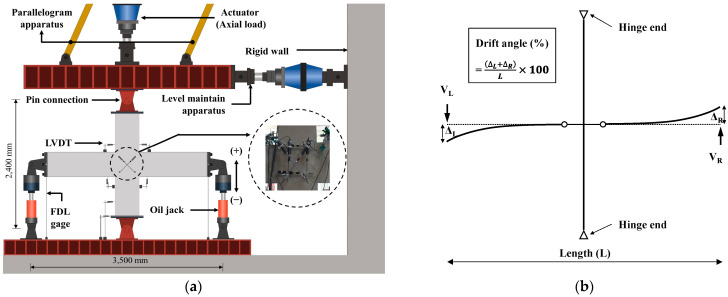
Test setup, instrumentation, and loading method. (**a**) Test setup; (**b**) Loading method (in positive direction).

Figure 3 shows the setup, instrumentation, and loading method. As shown in Figure 3a, the ends of the columns and beams were fixed using prefabricated hinges. The distances between the hinge centers of the columns and beams were 2400 mm and 3500 mm, respectively. Strain gauges were installed at the intersections of the columns and beams and at the plastic hinge areas of the beams to check the strain behavior of the cast iron reinforcement. In addition, six linear variable differential transducers (LVDTs) were attached to the joint by inserting four anchors to measure the deformation of the joints. The total displacement of the specimen was measured using four LVDTs.

The experiment was conducted using a structure testing machine (Smart Natural Space Research Center). The load-bearing method of the experimental body was applied to the beam load-bearing method, as shown in Figure 3b, referring to the study of Yang et al. [28]. In this experiment, the axial load was kept at about 10% (85 kN, 0.1*f_ck_*·*A_g_*) to perform emulation evaluation of the structural performance of the PC and RC experiments. Variables due to changes in the axial load acting on the column were not considered [29,30]. The beam preloading method is less likely to cause slippage at the column connection, and it is possible to generate a purely plastic hinge in the beam. The load was repeatedly applied upward (+) and downward (−) using 1000 kN cylinders installed at both free ends of the beam. The story drift angle was calculated as the sum of the displacements of the left·and right beams (Δ*_L_*, Δ*_R_*) divided by the total length of the beam (L). Until the yielding of the beam reinforcement, the loading protocol involved a 0.25% story drift angle for 1 cycle, a 1% angle for 2 cycles, and repeated loading for 2 cycles for each drift angle in the following order—1*θ_y_,* 2*θ_y_,* and 3*θ_y_*—based on the yield drift angle (*θ_y_*).

## 3. Test Results and Discussion

### 3.1. Crack Patterns and Story Shear–Drift Angle

**Figure 4:**
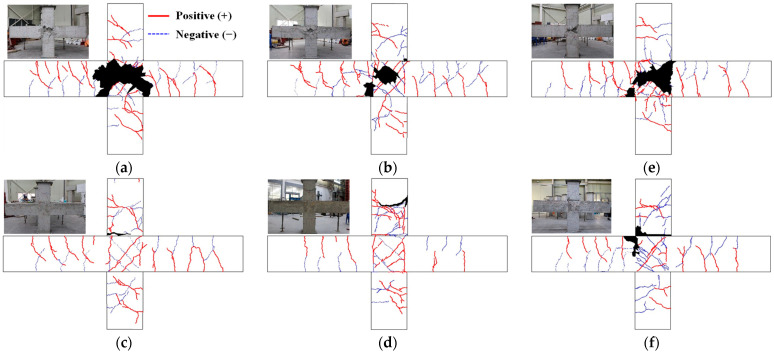
Cracking patterns of test specimens at end of test. (**a**) RC-BJ-I; (**b**) PC-BJ1-I; (**c**) PC-BJ2-I; (**d**) PC-BJ3-I; (**e**) RC-B-S; (**f**) PC-B-S.

**Figure 5:**
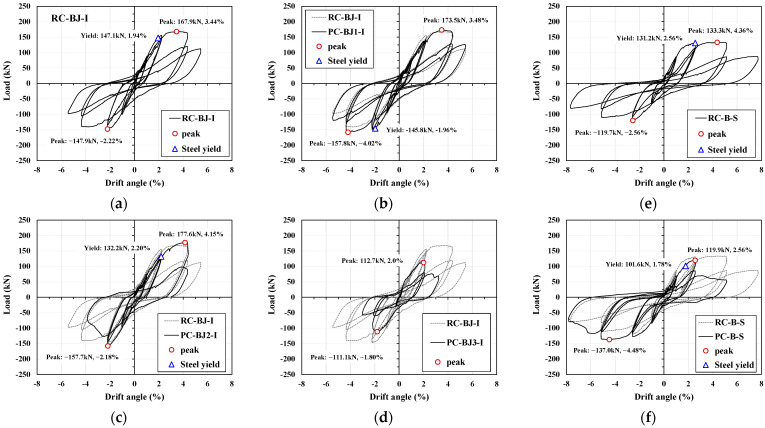
Load–drift angle hysteretic response. (**a**) RC-BJ-I; (**b**) PC-BJ1-I; (**c**) PC-BJ2-I; (**d**) PC-BJ3-I; (**e**) RC-B-S; (**f**) PC-B-S.

**Figure 6:**
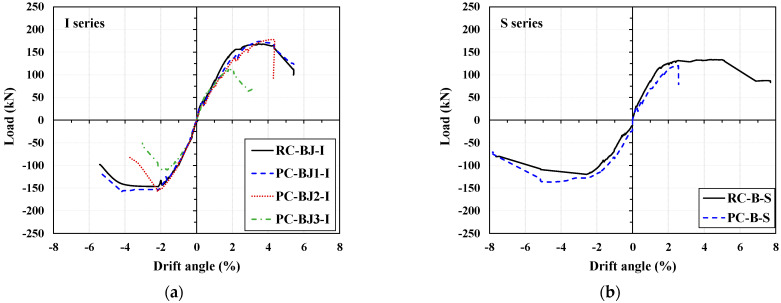
Skeleton curves of specimens. (**a**) I series; (**b**) S series.

Table 4 shows the experimental results, and Figure 4, Figure 5 and Figure 6 show the crack patterns, load story–drift angle relationships and skeleton curves of the experiments. For the I series, except for the PC-BJ2-I and PC-BJ3-I specimens, the specimens exhibited BJ failure, in which the joint fails after the longitudinal reinforcement yielding of the beam, as intended by the design. As shown in Figure 4a and Figure 5a, at low load levels, the RC-BJ-I specimen first developed flexural cracks in the plasticized hinge region. As the load increased, multiple diagonal cracks began to develop in the joints. The longitudinal reinforcement in the beam yielded at a load of 147.1 kN and a strain angle of 1.94%. The crack width of the joint increased accordingly and reached the maximum load at 167.9 kN and 3.44% strain, after which the load-resisting capacity of the specimen decreased rapidly. Diagonal cracks in the joints were clearly visible, but no diagonal cracks appeared in the plastic hinge area of the beam. The crack in only the plasticized hinge was a bending crack, as shown in Figure 4a. As shown in Figure 4b and Figure 5b, the PC-BJ1-I specimen also exhibited cracking patterns similar to those of the RC-BJ-I specimen, and the load (−145.8 kN) and strain (−1.96%) of the beam at yield and the peak load (173.5 kN) and strain (3.48%) were similar. However, the cracks and damage in the joints were lower than those of the RC-BJ-I test case because the splice sleeve connecting the upper and lower columns was filled with mortar, so there were no localized cracks or breaks near the sleeve. On the other hand, PC-BJ2-I exhibited behavior similar to that of RC-BJ-I during the forward load; however, after reaching the maximum load in the forward direction (177.6 kN, 4.15%), the load-bearing capacity rapidly decreased due to column reinforcement pullout due to poor mortar filling inside the splice sleeve of the column part, and the structure finally failed due to the sleeve adhesion failure. PC-BJ3-I was designed with a beam–column flexural resistance ratio of 1:1; however, due to insufficient concrete strength, the column’s resistance was lower than the design value, and it was finally destroyed by column failure at a lower load (112.7 kN) and deformation angle (2.0%) than those of the other experiments.

For the S series, the RC-B-S specimen exhibited cracking behavior similar to that of the RC-BJ-I specimen, as shown in Figure 4e and Figure 5e, but relatively fewer cracks occurred in the joints due to the low tensile reinforcement ratio of the beam.

The peak load (133.4 kN) was low, and the strain angle (4.36%) was relatively high. This is similar to previous experimental results related to the beam–column joints [31].

In comparison, the PC-B-S test case slipped after reaching a load of 119.9 kN at a strain angle of 2.56% during the application of forward force, just like the RC-BJ2-I test case, due to poor mortar filling inside the splice sleeve used to connect the reinforcing bars in the column. However, the negative lateral force was similar to that of RC-B-S.

### 3.2. Shear Stress-Strain

**Figure 7:**
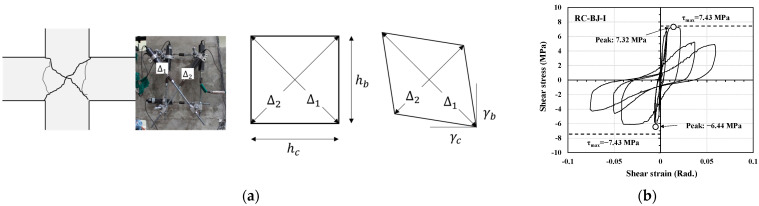

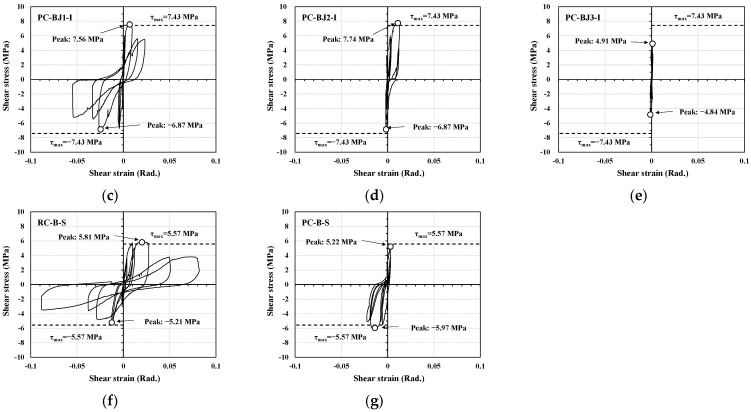
Beam–column joint area deformation. (**a**) Joint area deformation; (**b**) RC-BJ-I; (**c**) PC-BJ1-I; (**d**) PC-BJ2-I; (**e**) PC-BJ3-I; (**f**) RC-B-S; (**g**) PC-B-S.

To compare the shear behavior of the joints of each specimen, the shear stress and strain were calculated. As can be seen in Figure 7, the shear strain was calculated using Equation (3) with the value of the LVDT attached diagonally to the joint of the specimen [32,33]. The shear stress of the joint was calculated by dividing the joint horizontal shear force (*V_jby_*) at the yielding of the beam reinforcement, calculated using Equation (2), by the joint cross-sectional area, as shown in Equation (4).

The shear stress of the joint was calculated by dividing the horizontal shear force at the joint (*V_jby_*) at the yielding of the beam reinforcement, calculated using Equation (2), by the joint cross-sectional area.

(3) $$\gamma =\gamma_{b}+\gamma_{c}=\frac{\sqrt{h_{b}^{2}+h_{c}^{2}}}{2h_{b}h_{c}}\left(\Delta_{1}-\Delta_{2}\right)$$

(4) $$\tau =\frac{V_{jby}}{b_{j}h_{c}}$$

where *γ_b_* and *γ_c_* are the joint shear strain at the beam side and column side; *h_b_* and *h_c_* are the height and width of the joint core; Δ_1_ and Δ_2_ are deformations measured using dial indicators 1 and 2 (“+” for lengthening and “−” for shortening); and *b_j_* is the effective joint width defined by ACI 318-19 [25].

The joint shear strain values against the joint shear stress values of the specimens are shown in Figure 7. For the I series, as shown in Figure 7b,c, the shear stresses at maximum load were 7.32 MPa and 7.56 MPa for the BJ-failed RC-BJ-I and PC-BJ1-I specimens, respectively, which reached the design shear stress (7.43 MPa) as intended by the design. The shear strain values at this time were 0.014 rad for RC-BJ-I and 0.006 rad for PC-BJ1-I, this latter of which was approximately 40% lower. In addition, there was a difference in the shear strain of the joint at the end of the test (drift angle = 5.44%) after reaching the maximum shear stress. The RC-BJ-I specimen had a value of 0.059 rad, while the PC-BJ1-I specimen value had a value of 0.023 rad, the latter being about 40% lower, and showed a similar trend to the crack pattern. In comparison, the PC-BJ2-I and PC-BJ3-I experiments with the sleeve attachment failure and column failure showed lower shear strain values at the joint.

For the S series, as shown in Figure 7f, the RC-B-S specimen reached the design shear stress (5.57 MPa), with a shear stress of 5.81 MPa at peak load, after which the joint shear capacity decreased rapidly. The PC-B-S specimen exhibited a low level of shear deformation at the joint during upward (+) loading.

### 3.3. Strain Distribution

**Figure 8:**
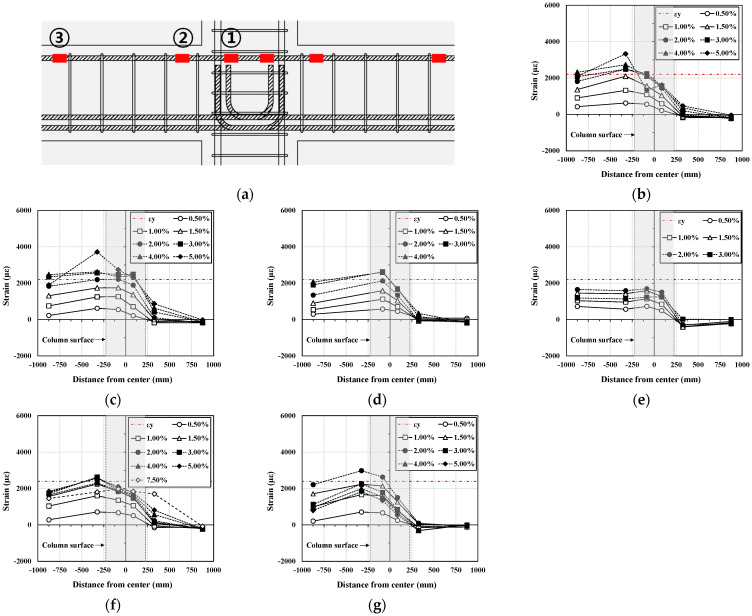
Comparison of strain distribution in beam longitudinal reinforcement. (**a**) Joint area deformation; (**b**) RC-BJ-I; (**c**) PC-BJ1-I; (**d**) PC-BJ2-I; (**e**) PC-BJ3-I; (**f**) RC-B-S; (**g**) PC-B-S.

Figure 8 shows the strain distribution in the longitudinal reinforcement of the beam as the drift angle increases. The figure also shows the yield strain of the reinforcement. In Figure 8, the strain of the upper longitudinal reinforcement of the beam gradually increased with an increase in the drift angle. In addition, all the specimens reached the yield strain except for the PC-BJ3-I specimen. Because the specimens were subjected to repeated loading, the strain of the lower reinforcement was almost identical to that of the upper reinforcement.

For the I series, as shown in Figure 8b, the RC-BJ-I specimen showed strain near the plastic hinge (②) of the beam, which increased with an increasing drift angle and was higher than the strain inside the joint (①). After the longitudinal tensile reinforcement of the beam yielded, as intended by the design, the strain inside the joint (①) dropped rapidly at a drift angle of 5% and the joint failed. As shown in Figure 8c, the PC-BJ1-I specimen exhibited a strain distribution similar to that of the RC-BJ-I specimen, but the difference between the strain near the plastic hinge (②) and the strain inside the joint (①) was not significant before the failure. For the S series, both RC-B-S (Figure 8f) and PC-B-S (Figure 8g) showed strain in the vicinity of the plastic hinge (②) of the beam, which increased with an increase in the drift angle, as in the I series, and was higher than the strain inside the joint (①).

### 3.4. Ductility Index and Energy Dissipation Capacity

**Figure 9:**
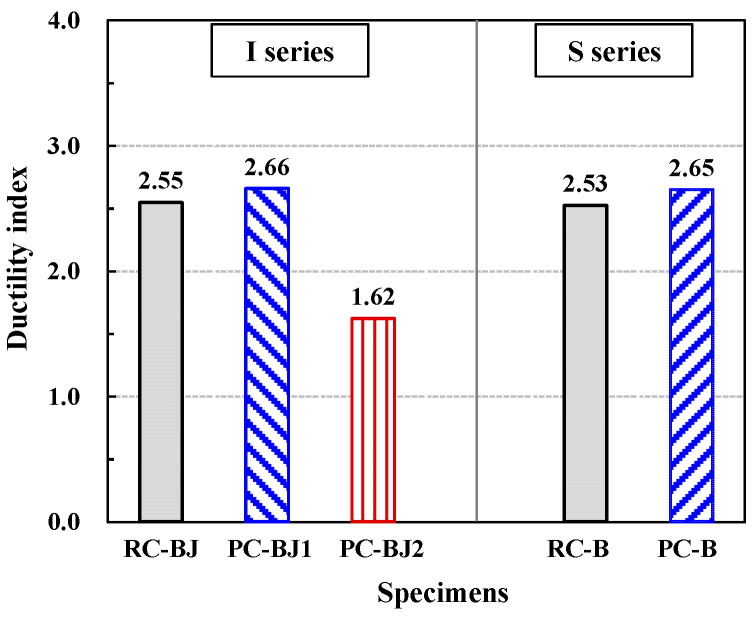
Comparison of ductility indexes of specimens.

The ductility index of the test specimens is shown in the 11th column of Table 4 and plotted in Figure 9. The ductility index was defined as the ratio of the displacement at the longitudinal reinforcement yielding of the beam to the displacement at 80% load reduction after reaching the peak load. The RC-BJ-I and PC-BJ1-I specimens of the I series exhibited similar ductility indexes of 2.25 and 2.66, respectively. The PC-BJ2-I specimen showed a lower ductility index of 1.62 due to the failure of the column reinforcement inside the splice sleeve. For the S series, RC-B-S and PC-B-S showed ductility indexes of 2.53 and 2.65, respectively, similar to those of the I series.

**Figure 10:**
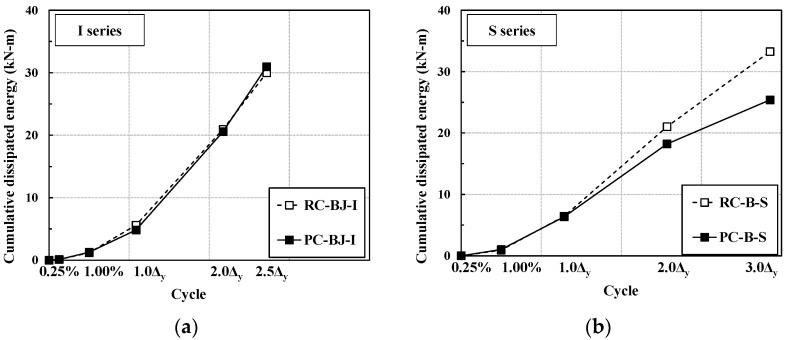
Comparison of cumulative energy dissipation. (**a**) I series; (**b**) S series.

The relationship of the cumulative energy dissipation (y axis) corresponding to one cycle of controlled displacement (x axis) is shown in Figure 10. For the I series, the RC-BJ-I and PC-BJ1-I experiments showed similar values of cumulative energy dissipation. For the S series, the RC-B-S and PC-B-S experiments showed similar values of cumulative energy dissipation until the beam longitudinal reinforcement yielded; however, after yielding, the PC-B-S experiment showed a lower level of energy dissipation due to the slipping of the column reinforcement inside the splice sleeve.

### 3.5. Emulation Evaluation of Specimens

In the study of Lee et al. [34], the equivalence criteria were evaluated using relative and absolute criteria. The relative evaluation criteria were used to directly compare the performance of the RC specimen with that of the PC specimen (AIJ [21]). The absolute evaluation criteria were used to determine the seismic performance of the PC specimen alone (ACI 374.1-05 [20]). For reinforced concrete structures, there is no distinction between intermediate and special moment-resisting frames based on the drift ratio. However, the American Institute of Steel Construction (AISC) [35] specifies a minimum drift ratio of 2.0% at the beam–column joints of intermediate moment-resisting frames. Therefore, Lee et al. [23] considered *θ* = 2.0% the equivalence criterion for PC intermediate moment-resisting frames, and *θ* = 3.5% that for special moment-resisting frames; these values are the same as those in the ACI 374.1-05 [20] guideline. The same criteria were applied in this paper.

**Figure 11:**
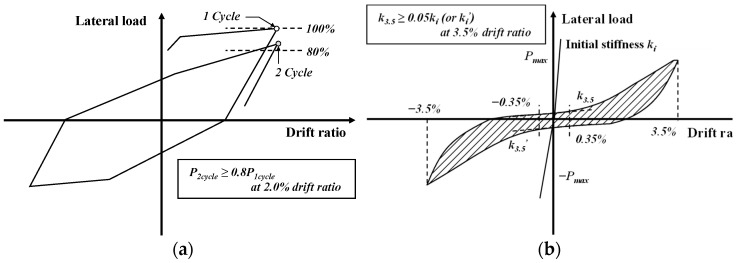
Emulation evaluation criteria. (**a**) Strength reduction (AIJ (2002) [21]) and (**b**) stiffness (ACI 374.1-05 [20]).

In the relative emulation evaluation, the yield strength, deformation at yield, and peak strength are shown in columns 3, 4, and 6 of Table 4. In addition, the strength reduction, calculated by referring to Figure 11a, is shown in Table 5. For the RC-BJ-I and PC-BJ1-I specimens of the I series, the yield strengths of the RC and PC specimens were similar at 147.1 kN and −145.8 kN, respectively, while the peak strength of the PC specimen (173.5 kN) was about 3% higher than that of the RC specimen (167.9 kN). The deformation at yield was similar at 34 mm for the RC and PC specimens. Furthermore, the strength reduction showed that the PC specimen met the relative equivalence guideline as the strength of the second cycle was more than 80% of that of the first cycle at a displacement ratio of 2.0%. In contrast, the S series PC-B-S specimens did not meet the relative evaluation criteria. For the absolute emulation evaluation, Table 5 shows the stiffness, calculated by referring to Figure 11b. The initial stiffness (*k_i_*) was measured by connecting the starting point with the angle of deflection at flexural yield (*θ_y_*). Both the I series (PC-BJ1-I) and S series (PC-B-S) specimens showed that the stiffness of the third hysteresis curve at a displacement ratio of 3.5% was more than 5% of the initial stiffness, satisfying the absolute evaluation criterion for stiffness.

## 4. Conclusions

In this study, using the PC method, a basic experimental study was conducted on the seismic performance of beam–column connection members for moment-resisting frames in moderate, weak, and strong seismic regions. The results of this study can be summarized as follows.

(1) As intended by the design, the load–drift angle relationship and crack pattern, shear stress–strain relationship, ductility index, and energy dissipation capacity of the fractured specimens showed that the PC specimens had a structural performance similar to that of RC specimens.
(2) In the cases of PC-BJ2-I and PC-B-S, the seismic performance decreased compared to that of RC due to the failure of the column reinforcement in the splice sleeve, confirming the importance of the mortar filling of the splice sleeve.
(3) In the I series, the relative evaluation criteria of yield strength, deformation at yield, and maximum strength showed a seismic performance more than equivalent to that of RC; the absolute evaluation criteria of stiffness was also satisfied.
(4) In the S series, the relative evaluation criteria showed a seismic performance lower than that of RC, while the absolute evaluation criterion, stiffness, showed satisfactory results.
(5) The emulation evaluation criteria for PC structures targeting special moment-resisting frames (strong seismic zones) confirmed the possibility of applying intermediate moment-resisting frames to target medium/weak seismic zones. However, further research is needed to examine the influence of the beam–column moment ratio.

## Figures and Tables

**Table 1:** Parameters of test specimens.

Specimens		Beam Joint Capacity Ratio			Beam–Column Capacity Ratio		
		*V*_*j*1_/*V_jby_*	*V*_*j*1_ (kN)	*V_jby_* (kN)	*M_c_*/*M_b_*	*M_c_* (kN·m)	*M_b_* (kN·m)
I series	RC-BJ-I	1.25	1669.5	1337.9	1.65	427.7	259.4
	PC-BJ1-I	1.25	1669.5	1337.9	1.65	427.7	259.4
	PC-BJ2-I	1.25	1669.5	1337.9	1.24	320.8	259.4
	PC-BJ3-I	1.25	1669.5	1337.9	0.98	253.2	259.4
S series	RC-B-S	1.66	1669.5	1002.9	1.63	320.8	196.6
	PC-B-S	1.66	1669.5	1002.9	1.63	320.8	196.6

Note: *V*_*j*1_: joint shear strength from ACI; *V_jby_*: shear on joint at beam yield; *M_c_*: column moment strength; *M_b_*: beam moment strength.

**Table 2:** Details of test specimen and material properties.

Moment-Resisting Frame	Specimen	*f_ck_* (MPa)	Column				Beam			
			Rebar		Hoop		Rebar		Stirrup	
			*f_cy_* (MPa)	*n_c_*	*f_h_* (MPa)	*n_h_*	*f_by_* (MPa)	*n_b_*	*f_s_* (MPa)	*n_s_*
I series	RC-BJ-I	35	439	8-D29	558	D10 @100	479	4-D22	558	D10 @150
	PC-BJ1-I	35	439	8-D29	558	D10 @100	479	4-D22	558	D10 @150
	PC-BJ2-I	35	439	6-D29	558	D10 @100	479	4-D22	558	D10 @150
	PC-BJ3-I	35	442	6-D25	558	D10 @100	479	4-D22	558	D10 @150
S series	RC-B-S	35	439	6-D29	558	D10 @100	448	4-D19	558	D10 @150
	PC-B-S	35	439	6-D29	558	D10 @100	448	4-D19	558	D10 @150

Note: I: intermediate moment-resisting frame, S: special moment-resisting frame; *f_ck_*: compressive strength of concrete; *f_cy_*, *f_h_*, *f_by_*, *f_s_*: yield strength of column rebar, hoop, beam rebar, and beam stirrup, respectively, *n_c_*: size of longitudinal steel bars in column, *n_h_*: size of hoop bar, *n_b_*: size of longitudinal steel bars in beam, *n_s_*: size of stirrup.

**Table 3:** Properties of reinforcing bars.

Rebar		f_y_* (MPa)	*ε_y_* (mm/mm)	*E_s_* (GPa)	Type of Specimens
Column longitudinal	D29	439	0.002384	189	RC-BJ-I, PC-BJ1-I, PC-BJ2-I, RC-B-S, PC-B-S
	D25	442	0.002384	185	PC-BJ3-I
Beam longitudinal	D22	480	0.002571	186	RC-BJ-I, PC-BJ1-I, PC-BJ2-I, PC-BJ3-I
	D19	448	0.002523	177	RC-B-S, PC-B-S
Transverse	D10	559	0.003023	185	All specimens

Note: f_y_*: 0.2% offset.

**Table 4:** Test result.

Specimen		*P_y_* (kN)	*θ_y_* (%)	Δ*_y_* (mm)	*P_peak_* (kN)	*θ_peak_* (%)	Δ*_peak_* (mm)	*θ*_0.8*peak*_ (mm)	Δ_0.8*peak*_ (mm)	Ductility Index
I series	RC-BJ-I	147.1	1.94	34.0	167.9 −147.9	3.44 −2.22	60.2 −38.9	4.90 −5.00	85.7 −87.6	2.55
	PC-BJ1-I	−145.8	−1.96	−34.0	173.5 −157.8	3.48 −4.02	60.9 −70.4	4.90 −5.43	85.8 −95.0	2.66
	PC-BJ2-I	132.2	2.20	38.5	177.6 −157.7	4.15 −2.18	72.6 −38.2	4.34 −2.79	76.0 −48.8	1.62
	PC-BJ3-I	-	-	-	112.7 −111.1	2.00 −1.80	35.0 −31.5	2.34 −2.92	40.9 −51.1	-
S series	RC-B-S	131.2	2.56	44.8	133.3 −119.7	4.36 −2.56	76.3 −44.8	6.07 −6.86	106.2 −120.0	2.53
	PC-B-S	101.6	1.78	31.2	119.9 −137.0	2.56 −4.48	44.8 −78.4	2.97 −6.48	52.0 −113.3	2.65

**Table 5:** Emulation evaluation of specimens.

Specimen		RC-BJ-I		PC-BJ1-I		RC-B-S		PC-B-S	
Loading Direction		(+)	(−)	(+)	(−)	(+)	(−)	(+)	(−)
Strength reduction, (kN) * (AIJ [21])	P_2.0_, 1 cycle	149.9		136.2		125.2		112.2	
	0.8P_2.0_, 1 cycle	119.9		109.0		100.2		89.8	
	P_2.0_, 2 cycle	135.1		135.6		89.8		74.2	
Stiffness, (kN/mm) ** (ACI 374.1-05 [20])	k_i_	4.34	4.21	3.96	4.24	2.93	2.67	3.26	3.65
	0.05k_i_	0.22	0.21	0.20	0.21	0.15	0.13	0.16	0.18
	K_3.5_	1.78	1.41	1.80	1.64	1.04	0.84	1.07	2.13

Note: * Refer to Figure 11a, ** refer to Figure 11b.

## Data Availability

The data presented in this study are available upon request from the corresponding author.
